# PERK silence inhibits glioma cell growth under low glucose stress by blockage of p-AKT and subsequent HK2's mitochondria translocation

**DOI:** 10.1038/srep09065

**Published:** 2015-03-12

**Authors:** Xu Hou, Yaohua Liu, Huailei Liu, Xin Chen, Min Liu, Hui Che, Fei Guo, Chunlei Wang, Daming Zhang, Jianing Wu, Xiaofeng Chen, Chen Shen, Chenguang Li, Fei Peng, Yunke Bi, Zhuowen Yang, Guang Yang, Jing Ai, Xin Gao, Shiguang Zhao

**Affiliations:** 1 Department of Neurosurgery, First Affiliated Hospital of Harbin Medical University, Harbin, Heilongjiang Province, People's Republic of China; 2 College of Basic Medicine, Beijing University of Chinese Medicine, Beijing, People's Republic of China; 3 Department of Pharmacology (State-Province Key Laboratories of Biomedicine-Pharmaceutics of China), Harbin Medical University, Harbin, Heilongjiang Province, People's Republic of China; 4 Department of Endocrinology, First Affiliated Hospital of Harbin Medical University, Harbin, Heilongjiang Province, People's Republic of China; 5 Computer, Electrical and Mathematical Sciences and Engineering Division, King Abdullah University of Science and Technology (KAUST), Thuwal, Kingdom of Saudi Arabia

## Abstract

Glioma relies on glycolysis to obtain energy and sustain its survival under low glucose microenvironment *in vivo*. The mechanisms on glioma cell glycolysis regulation are still unclear. Signaling mediated by Double-stranded RNA-activated protein kinase (PKR) – like ER kinase (PERK) is one of the important pathways of unfolded protein response (UPR) which is comprehensively activated in cancer cells upon the hypoxic and low glucose stress. Here we show that PERK is significantly activated in human glioma tissues. PERK silencing results in decreased glioma cell viability and ATP/lactate production upon low glucose stress, which is mediated by partially blocked AKT activation and subsequent inhibition of Hexokinase II (HK2)'s mitochondria translocation. More importantly, PERK silenced glioma cells show decreased tumor formation capacity. Our results reveal that PERK activation is involved in glioma glycolysis regulation and may be a potential molecular target for glioma treatment.

Glioma cells grow in a hypoxic and low glucose microenvironment *in vivo*, but need to uptake plenty of glucose to maintain their survival^1^,^2^. The microenvironmental stress makes glioma cells obtain enough energy via their own particular metabolism and gene expression. The shift from oxidative phosphorylation (OXPHOX) to glycolysis is the classic glucose metabolism style which promotes glioma cells to adapt to the microenviroment^3^. Although the recognition of the roles of different enzymes, such as Isocitrate dehydrogenase 1 (IDH1)^4^, HK2^5^ and Pyruvate kinase M2 (PKM2)^6^, regulating glucose metabolism helps scientists to better understand the metabolic characteristics of glioma, the molecular mechanisms for glioma metabolic regulation are still not clear.

PERK signaling is one of the important downstream pathways of UPR which is comprehensively activated in cancer cells upon the hypoxic and low glucose stress^7^,^8^. Numerous studies demonstrate UPR pathways play critical roles for cancer cell survival and tumor growth *in vivo* via activating adaptive strategies against the microenviromental stress^9^,^10^. Blockage of UPR signals including PERK arm leads to tumor growth arrest and cancer cell apoptosis in different malignancies^11^,^12^, which suggests that PERK is a potential molecular target for cancer therapy. Considering the tight cross-relationship among UPR signals, cancer microenvironmental stress and energy metabolism, it is reasonable to speculate that UPR molecules may be involved in cancer cell metabolism regulation. Recent years increasing evidence shows that PERK confers great impacts on cell metabolism in metabolic disorders^13^,^14^. PERK is clearly activated upon low glucose stress, and inhibition of PERK converts tumor cells from growth inhibition to cell death^7^. However, the role of PERK in human glioma is not clear.

In human glioma, AKT is highly activated by its form of phosphorylation at Ser473, which promotes glioma cells proliferation, migration and provides resistance against apoptosis^15^,^16^,^17^. Inhibition of AKT phosphorylation by its inhibitors which have been used in I and II clinical trials is considered as a promising way for cancer treatment^18^,^19^. More importantly, p-AKT is a crucial modulator of glucose metabolism in different cells^20^,^21^. To be specific, in human glioma, the role of p-AKT on glycolysis is mainly via its regulation on the mitochondria translocation of HK2, which is a key enzyme for glioma energy metabolism and necessary for cell survival under metabolic stress, to mitochondria in glioma cells, resulting in OXPHOX's inhibition and a large amount of lactate production^5^.

Recently, several studies have demonstrated that PERK activation was tightly related to AKT phosphorylation in kinds of cells^22^,^23^,^24^. Some researchers reported that PERK activation was directly regulated by AKT^23^,^24^. More importantly, some evidence showed that AKT was phosphorylated by a PERK-dependent way at Ser-473 during ER stress^22^. It is interesting to explore the relationship of these two important pathways both of which are key regulators for cancer cell survival. Whether PERK phosphorylation under the microenviromental stress in glioma tissues may stimulate glycolysis via the regulation of AKT pathway on HK2 remains unclear.

In this study, for the first time, we showed that PERK was significantly activated in glioma tissues. Moreover, PERK silencing inhibited glioma cell viability and tumor growth by blocking AKT phosphorylation and consequently disrupting HK2's mitochondria translocation and glycolysis under low glucose metabolism stress. Those data suggested PERK may be a molecular target for glioma treatment.

## Results

### PERK is strongly activated in glioma tissues

**Figure 1:**
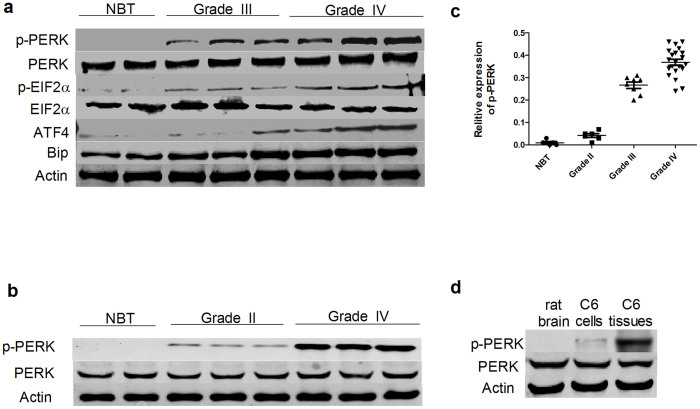
PERK is strongly activated in glioma tissues. (a–b) Westernblot analyses of p-PERK, PERK, p-EIF2α, EIF2α, Bip and ATF4 protein expression in different grades of gliomas and nontumor brain tissues (NBT). (c) p-PERK relative protein of 35 human gliomas (WHO grade II = 6,WHO grade III = 8, and WHO grade IV = 21) and 5 nontumor brain tissues expression level were normalized to PERK. (d) Westernblot analyses of p-PERK and PERK protein expression in rat brain tissues, C6 cells cultured *in vitro* and intracerebral glioma tissues formed by C6 cells.

PERK activation is an important marker of UPR and protects cancer cells against different kinds of stress such as hypoxia and nutrients deprivation inside solid tumors *in vivo*^7^,^8^. However, little is known about its effects in glioma. In our study, PERK signaling pathway related proteins were first examined in glioma tissues. Westernblot analysis results showed that UPR related indicators including p-PERK, immunoglobulin heavy chain binding protein (Bip), activating transcription factor 4 (ATF4), and phospho-eukaryotic initiation factor 2α (p-EIF2α) were all highly upregulated in anaplastic astrocytomas (WHO grade III) and glioblatomas (WHO grade IV) compared with nontumor brain tissues, while there was no significant difference of PERK protein level among them (Figure 1 a and Supplementary figure 1). Although there was strong increase of p-PERK in high grade glioma tissues (WHO grade III and IV), the p-PERK level (Figure 1 b) was only slightly elevated in astrocytomas (WHO grade II), which suggested a possible positive correlation between p-PERK level and malignant degree in gliomas (Figure 1 c and Supplementary figure 2), while such a tendency needed to be further confirmed in larger number of tissue samples. Considering the tumor cell proliferation was much more rapid in high grade gliomas, which lead to a more decreased oxygen and glucose level, it was reasonable that PERK activation would be much stronger in high grade gliomas. To further investigate the effect of *in vivo* microenviromental stress on PERK activation, we compared the p-PERK level in C6 glioma cells *in vitro* and *in vivo*. The results showed that PERK was unphosphorylated in normal brain tissue and C6 cells cultured *in vitro* while clearly activated in C6 intracranial glioma tissues (Figure 1 d).

### PERK silencing suppressed glioma cell viability and induced apoptosis under low glucose metabolism stress

**Figure 2:**
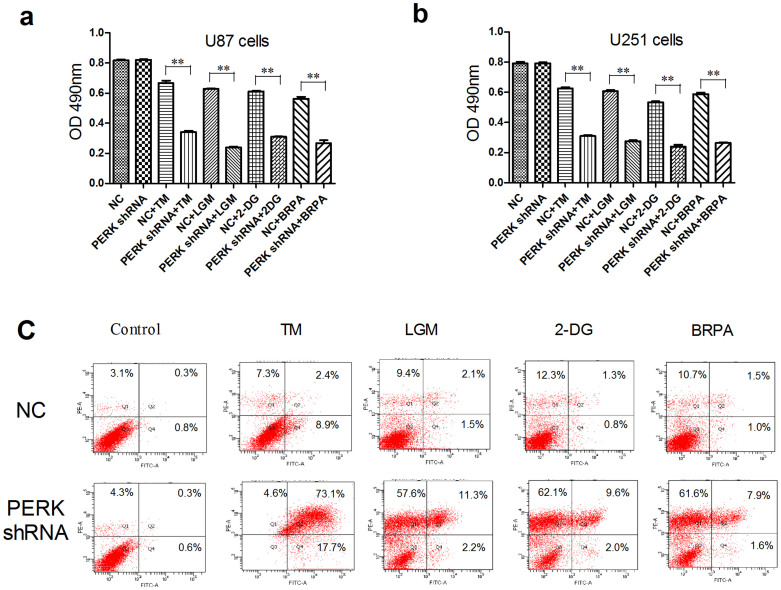
PERK silencing suppressed glioma cell viability and induced apoptosis under low glucose metabolism stress. (a–b) U87 and U251 cells were transfected with NC or PERK shRNA, and then 48 hours later the cells were treated by TM, 2-DG, BRPA or grown in low glucose medium respectively for 48 h. Cell viability was analysed by MTT (OD = 490 nm). The results repeated three times and the data shown represent mean ± SEM of three independent experiments. **, P < 0.01. (c) U87 cells were transfected with NC or PERK shRNA, and then 48 hours later the cells were treated by TM (ER stress inducer), 2-DG, BRPA or grown in low glucose medium respectively for 48 h. Cell apoptosis was analyzed by flow cytometry analysis.

In order to clarify biological function of PERK activation in glioma cells, we mimicked low glucose microenvironment in glioma tissues by culturing glioma cells in low glucose medium (LGM), or in DMEM with 2-deoxy-D-glucose (2-DG) or bromopyruvic acid (BRPA) which are inhibitors of glycolysis and can effectively disrupt glucose utilization^25^,^26^,^27^,^28^. Tunicamycin (TM) was used as a UPR inducer for positive control. As shown in Figure 2 a–b, TM treatment clearly inhibited cell viability in PERK silenced cells compared with that in negative control (NC) cells, which was reasonable since UPR pathway was impaired after PERK knockdown. Although inhibition of PERK alone had little effect on glioma cell viability, PERK silencing effectively inhibited cell viability under LGM or treated by 2-DG or BRPA. Importantly, flow cytometry analysis showed that the rate of apoptosis was elevated in PERK shRNA transfected cells versus NC transfected cells under LGM culture microenvironment or the DMEM treated by 2-DG or BRPA (Figure 2 c). In all, PERK inhibition strongly decreased cell viability and induced apoptosis under low glucose metabolism stress.

### PERK silencing decreased p-AKT in glioma cells under low glucose metabolism stress

**Figure 3:**
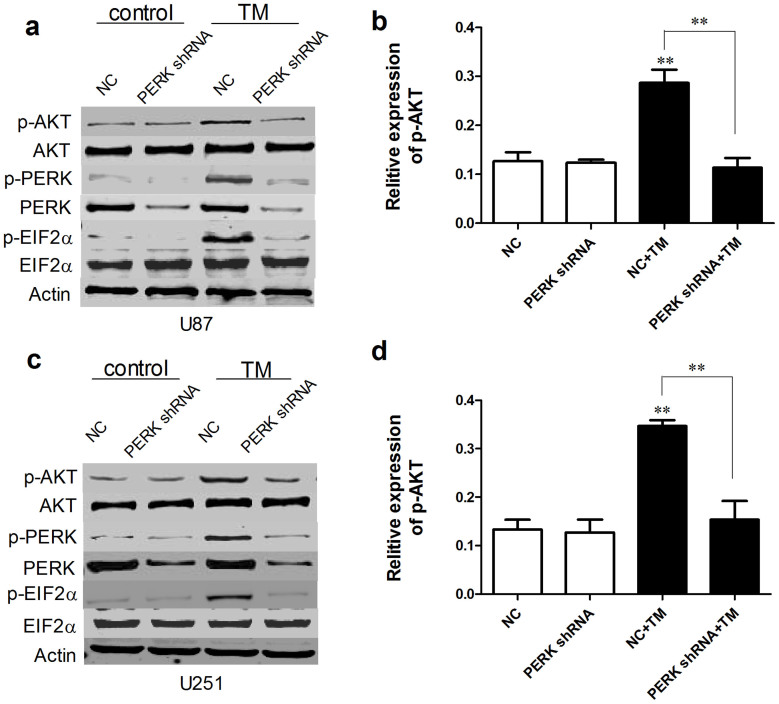
PERK silencing decreased p-AKT in glioma cells under ER stress. U87 and U251 cells were transfected with NC or PERK shRNA and 48 hours later the cells were treated by TM (ER stress inducer) for another 48 h. Western-blot analyses were performed to detect p-AKT (Ser473), AKT, PERK, p-PERK, p-EIF2α and EIF2α protein expression. Statistical analysis of p-AKT/AKT was derived from 3 independent experiments which were run under same conditions. **, P < 0.01.

**Figure 4:**
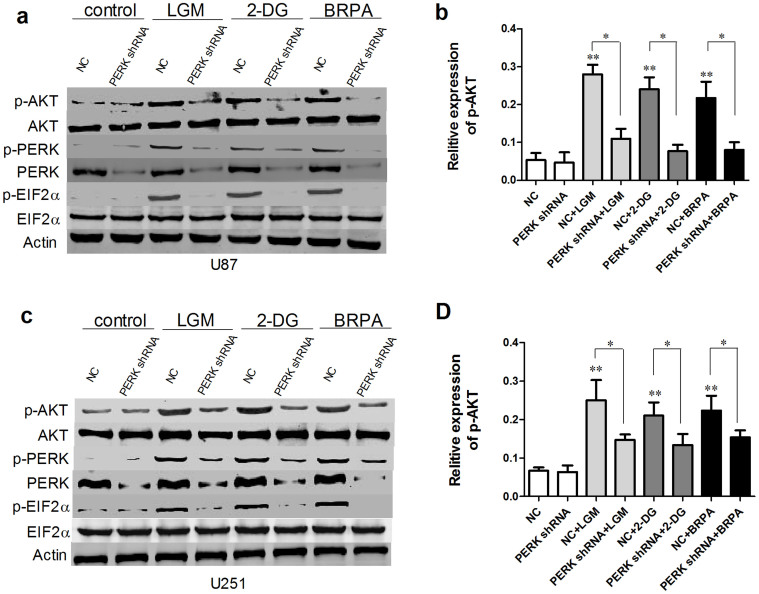
PERK silencing decreased p-AKT in glioma cells under low glucose metabolism stress. U87 and U251 cells were transfected with NC or PERK shRNA and then were treated by 2-DG, BRPA or grown in LGM for 48 h. Westernblot analyses were performed to detect p-AKT (Ser473), AKT, PERK p-PERK, p-EIF2α and EIF2α protein expression. Statistical analysis of p-AKT/AKT was derived from 3 independent experiments which were run under same conditions. *, P < 0.05, **, P < 0.01.

Owing to the tight relationship between PERK phosphorylation and AKT activation in kinds of cells^22^,^23^, we investigated the expression level of p-PERK and p-AKT (Ser473) in glioma cells. We first examined p-PERK and p-AKT protein level in PERK silenced glioma cells in presence of TM. As shown in Figure 3, p-PERK and p-AKT induced by TM were both clearly inhibited in PERK shRNA transfected cells compared with NC transfected cells. Then we detected p-AKT protein level in glioma cells transfected with NC or PERK shRNA under normal condition, LGM or glucose utilization disrupting agents treatment by Westernblot analysis. Figure 4 showed that low glucose medium, 2-DG and BRPA treatment all activated PERK and AKT phosphorylation, while interestingly, AKT phosphorylation level was strongly blocked by PERK silencing. In all, these results indicated that PERK silencing effectively inhibited AKT phosphorylation under ER stress or low glucose metabolism condition in glioma cells.

### EGF rescued p-AKT level and cell viability in PERK silenced glioma cells under low glucose metabolism stress

**Figure 5:**
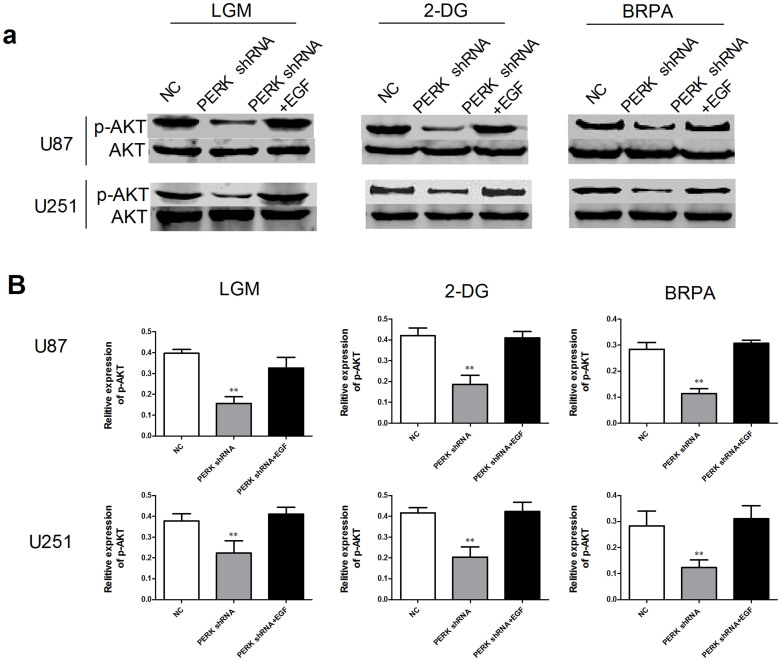
EGF rescued p-AKT level in PERK silenced glioma cells under low glucose metabolism stress. U87 and U251 cells were transfected with NC or PERK shRNA, then 48 hours later the cells were treated under LGM or incubated by 2-DG or BRPA for 48 hours with or without pretreatment of EGF for 6 hours. (a) Westernblot analyses were performed to detect p-AKT protein expression. (b) Statistical analysis of p-AKT/AKT was derived from 3 independent experiments which were run under same conditions. **, P < 0.01.

**Figure 6:**
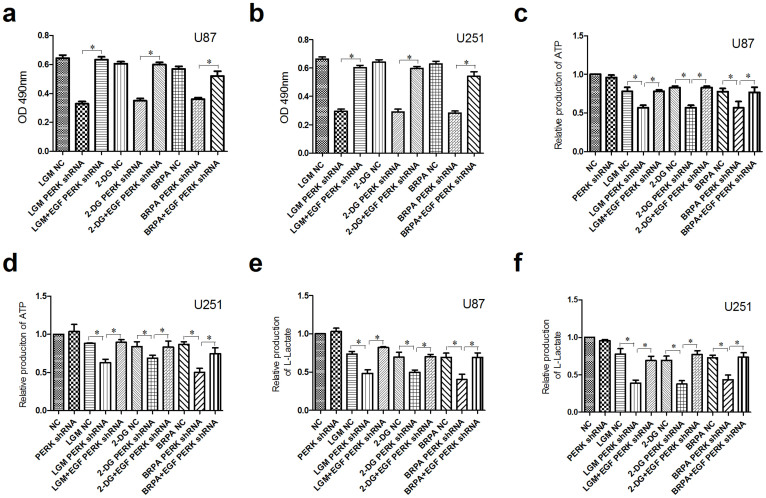
EGF rescued cell viability in PERK silenced glioma cells under low glucose metabolism stress. U87 and U251 cells were transfected with NC or PERK shRNA, then 48 hours later the cells were treated under LGM or incubated by 2-DG or BRPA for 48 hours with or without pretreatment of EGF for 6 hours. (a–b) Cell viability was analyzed by MTT. (c–d) ATP production was examined by ATP kit. (e–f) Lactate production was examined by lactate kit. The results repeated three times and the data shown represent mean ± SEM of three independent experiments. *, P < 0.05.

To further investigate the role of p-AKT on cell viability regulated by PERK silencing under low glucose metabolism stress, one of the activators of AKT phosphorylation, EGF^5^,^29^, was administered to restore the p-AKT level induced by low glucose metabolism stress in PERK silenced glioma cells. Our results showed that EGF actually rescued the impaired p-AKT level (Figure 5), and more importantly, reversed cell viability (Figure 6 a–b) in PERK silenced cells under low glucose metabolism stress. These results were consistent with a variety of previous studies which indicated that PERK silencing inhibited AKT activation in kinds of cells^22^,^23^. Due to the fact that p-AKT is associated with glycolysis of glioma cells^5^, we then examined the effects of PERK inhibition on ATP and lactate production. As shown in Figure 6 c–f, although PERK blocking alone didn't affect ATP and lactate production, it clearly enhanced the decrease of ATP and lactate production mediated by LGM, 2-DG or BRPA, which can be effectively rescued by EGF.

### PERK silencing suppressed translocation of HK2 to mitochondria

**Figure 7:**
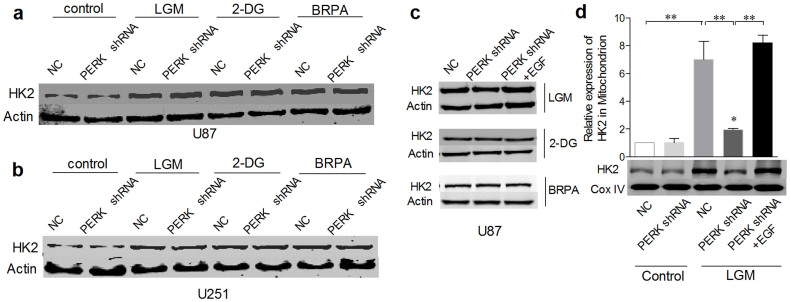
PERK silencing suppressed translocation of HK2 to mitochondria. (a–b) Westernblot analyses were performed to detect HK2 total protein expression after U87 and U251 cells were transfected with NC or PERK shRNA for 48 hours followed by treatment of 2-DG, BRPA or low glucose medium respectively for 48 h. (c) U87 cells were transfected with NC or PERK shRNA for 48 hours followed by LGM incubation, 2-DG or BRPA treatment for 48 hours with or without pretreatment of EGF for 6 hours. HK2 total protein was analysed by Westernblot. (d) U87 cells were transfected with NC or PERK shRNA for 48 hours followed by LGM incubation for 48 hours with or without pretreatment of EGF for 6 hours. Mitochondrial protein was extracted and HK2 expression was detected by Westernblot analyses and normalized to Cox IV. Statistical analysis of p-AKT/AKT was derived from 3 independent experiments which were run under same conditions. *, P < 0.05, **, P < 0.01.

It is well known that cancer cells utilize glucose metabolism for energy production mainly via glycolysis. Previous study showed that HK2 was induced by metabolic stress in glioma cells, furthermore, the binding of HK2 to mitochondria outer membrane, which was essential for glioma cell glycolysis, relied on PI3K-AKT pathway^5^. Our results demonstrated that low glucose metabolism stress clearly induced HK2 expression, while PERK silencing had no effects on its total induction level (Figure 7 a, b) and EGF administration didn't further increase HK2's total expression (Figure 7 c). Since HK2's role on glycolysis relies on its binding to mitochondria outer membrane, we then investigated HK2's mitochondria location. It is shown that LGM treatment induced clear elevation of HK2's level on mitochondria, which was strongly blocked by PERK silencing and rescued by EGF treatment in PERK silenced cells (Figure 7 d). Due to the fact that EGF is one of the important ligands leading to activation of PI3K–AKT pathway, cell viability and ATP/lactate production (Figure 6) rescued by EGF in PERK silencing cells under low glucose stress should be attributed by the EGF-restored AKT phosphorylation and subsequent recovery of HK2's mitochondria location.

### PERK silencing suppressed glioma growth in vivo

**Figure 8:**
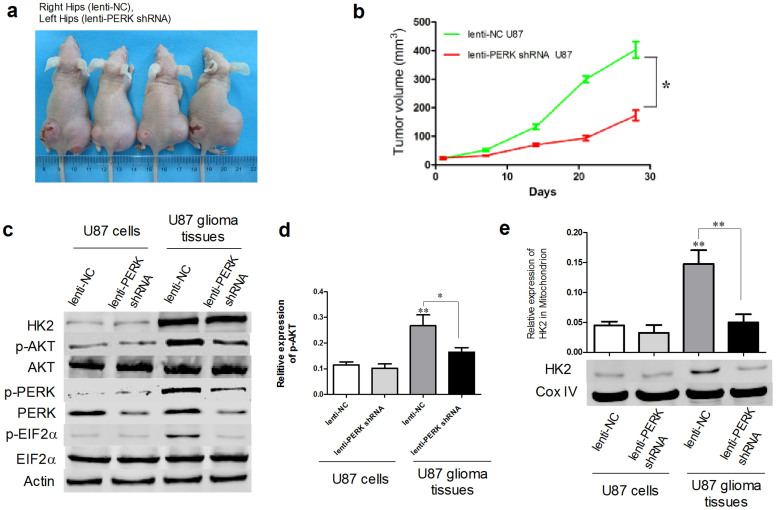
PERK silencing suppressed glioma growth *in vivo*. (a) lenti-PERK shRNA and lenti-NC U87 cells were subcutaneously injected into left and right hips of BALB/c nude female mice (n = 4). The typical images of mice bearing glioma were pictured 4 weeks later. (b) Tumor volumes were measured once a week and data was presented as mean ± SEM. *, P < 0.05. (c) Westernblot analysis of total HK2, p-AKT, p-PERK and p-EIF2α in indicated cells or glioma tissues respectively. (d) Statistical analysis of p-AKT/AKT was derived from 4 independent experiments. *, P < 0.05, **, P < 0.01. (e) Westernblot analyses of mitochondrial HK2 protein expression in indicated cells or glioma tissues respectively. Statistical analysis of mitochondrial HK2 protein expression was derived from 4 independent experiments. **, P < 0.01.

The above results indicated that PERK gene silencing significantly inhibited glioma cell viability, reduced ATP and lactate production under low glucose metabolism stress via decreasing AKT phosphorylation and further suppressing translocation of HK2 to mitochondria. It is known that glioma cells can survive under *in vivo* low glucose microenvironment inside tumors, thus PERK silencing may inhibit glioma growth *in vivo*. Our results indicated that lenti-PERK shRNA U87 glioma cells formed much smaller volume of glioma than lenti-NC glioma cells (Figure 8 a–b). Interestingly these smaller tumors formed by lenti-PERK shRNA glioma cells displayed ulcerations which were normally due to necrosis of rapidly proliferating cells. The possible reason for ulcerations may be that lenti-PERK shRNA derived tumors cannot tolerate the stress condition *in vivo* such as low glucose. We further investigated the interested proteins expression in these tumors. Westernblot results showed that tumors formed by lenti-shRNA PERK glioma cells had much lower p-AKT level than those formed by lenti-NC glioma cells (Figure 8 c–d). Although there is no obvious difference of total HK2 expression, the HK2 expression in mitochondria strongly decreased in tumors formed by lenti-shRNA PERK glioma cells compared with lenti-NC gliomas (Figure 8 c, e). Our results suggested that PERK played an important role on glioma carcinogenesis by regulation of AKT activation and subsequent HK2's mitochondria location.

## Discussion

UPR is extensively activated in cancers, which is an important adaptation for cancer cell survival against microenvironmental stress, such as hypoxia and nutrients deprivation^7^,^8^. Previous studies showed that blockage of PERK signaling, one of the three arms of UPR, resulted in increased susceptivity of cancer cells to hypoxia and metabolic stress *in vitro* and tumor growth retardation *in vivo*^11^,^12^. However, little is known about the role of UPR in human glioma. Our study for the first time demonstrated that PERK was clearly activated in human glioma tissues compared with nontumor brain tissues, more importantly, PERK silencing in glioma cells inhibited tumor growth *in vivo*, which suggested that PERK activation may play an important role for human glioma development.

The role of PERK in cancers is still unclear. Some studies revealed that PERK pathway activated autophagy and other protective signals in cancer cells upon stress, thus prevented cell apoptosis and promote the progression^12^,^30^,^31^. Cancers including glioma survive under the condition of low glucose and nutrients starvation due to the fast tumor growth and lack of blood supply^2^. Numerous studies showed that low glucose stress made the cancer cells more dependent on glycolysis for energy generation and drived tumors toward a more malignant style^32^,^33^,^34^. In our study, we found that PERK silencing clearly inhibited cell viability and ATP/lactate production under low glucose metabolism stress, which implied that PERK was really involved in glioma glycolysis regulation.

Glucose metabolism in glioma is mainly regulated by glycolytic enzymes upregulated under low glucose metabolism stress^5^,^20^,^35^,^36^. HK2 is the first key enzyme for glycolysis and is abundantly expressed in gliomas, moreover, HK2's expression level is negatively correlated with glioma (WHO grade IV) patients' prognosis^5^. HK2's function is dependant on AKT activation, upon which HK2 undergoes translocation to the outer membrane of mitochondria, leading to a metabolism shift from OXPHOS to glycolysis thus promoting cancer cell survival under metabolic stress^5^. Both AKT and PERK are important regulators for cancers. Previous studies revealed that AKT phosphorylation can be regulated by PERK activation^13^,^14^,^37^,^38^,^39^. In this paper, we also demonstrated that PERK silencing really blocked p-AKT and subsequently inhibited HK2's mitochondrial translocation and cell viability in glioma cells under low glucose stress. Some authors also suggested that PERK was a direct target of AKT whose phopshorylation could activate or inactivate PERK^23^. Thus possibly PERK and AKT can be regulated by each other. Our data suggested that AKT pathway activated by PERK under stress conditions may act as a modulator to alleviate the cellular stress level by initiating some protective behaviors such as glycolysis. In all, our results showed that PERK activation was tightly related to AKT drived glioma cell metabolism regulation.

In summary, for the first time, we revealed that PERK was activated in glioma and PERK silencing reduced glioma cell viability under low glucose stress in culture and suppressed glioma growth *in vivo* via inhibition of p-AKT and glioma cell glycolysis. Whether PERK silencing may act as a promising way for human glioma treatment by regulation of glucose metabolism is worthy of further investigation.

## Methods

### Patients samples collection

35 human gliomas (astrocytoma, WHO grade II = 6; anaplastic astrocytoma, WHO grade III = 8; glioblastoma, WHO grade IV = 21) and 5 nontumor brain tissues from the temporal lobes of five epileptic patients were collected from the Department of Neurosurgery, the First Affiliated Hospital of Harbin Medical University. All of the tissues were frozen in liquid nitrogen immediately after surgery removal and stored at −80°C for use. Informed agreement was obtained from all patients. This study was performed with ethical approval of the Human Ethics Committee of The First Affiliated Hospital of Harbin Medical University in accordance with the Declaration of Helsinki, and written informed consents were obtained from all of the enrolled subjects.

### Cell culture and MTT assay

C6, U87 and U251 cell lines were obtained from Cell Resource Center, IBMS, CAMS/PUMC where they were characterized by mycoplasma detection, short tandem repeat (STR) and cell vitality detection. These cell lines were immediately expanded and frozen such that they could be restarted every 3 to 4 months from a frozen vial of the same batch of cells. Cells cultured in DMEM high glucose medium (Invitrogen, USA) with 10% fetal bovine serum (Invitrogen, USA), 1% streptomycin/penicillin solution (Beyotime, China), at 37°C with 5% CO_2_. Cells were treated by Tunicamycin (TM, 2 g/L, Sigma, USA),2-deoxy-D-glucose(2-DG, 25 mM, Sigma, USA), Bromopyruvic acid (BRPA, 30 μM, Sigma, USA), Epidermal growth factor (EGF, 100 ng/ml, Peprotech, USA) or grew in low glucose medium (LGM, 2 mM). Cell viability was detected according to MTT assay kit's instructions (5 mg/mL, Sigma, USA).

### Westernblot analysis

Cell and tissue total proteins were collected with RIPA lysis buffer containing protease and phosphatase inhibitors (Roche, Swiss) according to the manufacturer instructions. Mitochondrial proteins were acquired with mitochondrial protein extracted kit (Sangon, China) according to manufacturer's protocol. Western blot was achieved as previously described. Membranes were incubated with the following antibodies: anti-PERK from Santa Cruz Biotechnology Inc. (Cat no. sc-13073, 1:100, Santa Cruz, CA, USA), anti-p-PERK(Thr981) from Santa Cruz Biotechnology Inc. (Cat no. sc-32577, 1:100, Santa Cruz, CA, USA), anti-p-EIF2α (Ser52) from Santa Cruz Biotechnology Inc. (Cat no. sc-101670, 1:100, Santa Cruz, CA, USA), anti-EIF2α from Santa Cruz Biotechnology Inc. (Cat no. sc-11386, 1:100, Santa Cruz, CA, USA), anti-ATF4 from Santa Cruz Biotechnology Inc. (Cat no. sc-200, 1:100, Santa Cruz, CA, USA), anti-Bip from Santa Cruz Biotechnology Inc. (Cat no. sc-33757, 1:100, Santa Cruz, CA, USA), anti-HK2 from Cell Signaling Technology (Cat no. #2867, 1:1000, Beverly, MA, USA), anti-COX IV from Cell Signaling Technology (Cat no. #4850, 1:1000, Beverly, MA, USA), anti-AKT from Cell Signaling Technology (Cat no. #4685, 1:1000,Beverly, MA, USA), anti-p-AKT(Ser473) from Cell Signaling Technology (Cat no. #4060, 1:1000,Beverly, MA, USA), and anti-β-ACTIN from Santa Cruz Biotechnology Inc. (Cat no. sc-8432, 1:100, Santa Cruz, CA, USA). Protein expression levels were evaluated by quantified gray density of Western-blot bands with Odyssey V1.2 software and normalized to internal controls.

### Cell infection and construction of lentiviral vector

PERK and negative control (NC) shRNAs were first constructed according to effective PERK's siRNA (5′ GCAGGUCAUUAGUAAUUAU 3′) and NC sequences (5′ UUCUCCGAACGUGUCACGU 3′) which were confirmed in this study. PERK and negative control (NC) shRNAs were used for transient transfection experiments in this study. For stable transfection, PERK and NC shRNAs were embedded into pENTR/U6-GFP vector with GFP, then recombination reaction was processed between pENTR/U6-shRNA-GFP and pLenti6/Block-it-DEST vector, at last, the combined vector and package plasmid were co-transfected into HEK 293T cells and then virus particles were collected after 48 h. U87 cells were then infected with lenti-PERK shRNA or NC vector. U87 cells which expressed GFP were chose for further culture.

### ATP and lactate detection

ATP and Lactate production were respectively detected by ATP kit (Beyotime, China) and Lactate assay kit (Eton Bioscience, San Diego, USA) on the basis of manufacturer's instructions and normalized to glioma cell numbers.

### *In vivo* tumor formation

C6 cell suspensions (containing 5 × 10^5^ cells) were injected at a depth of 5.0 mm into the caudate nucleus of 3 male Wistar rats weighing 200–250 g (Vital River Laboratory, Beijing, China). Rats were sacrificed at 21-day after C6 cell transplantation. At the same time, glioma and normal adjacent tissues were dissected and immediately frozen at −80°C for use. Four female BALB/c nude mice used in this study (4 weeks old) were purchased from Slac Laboratory Animal Company, Shanghai, China. 2 × 10^6^ lenti-NC or lenti-PERK shRNA stable transfected glioma cells were respectively injected into right and left hips of nude mice subcutaneously. Tumor dimensions were measured once a week with calipers and tumor volumes were calculated as width^2^ × length × 1/2. The mice were then sacrificed at 28-day. The glioma tissues were immediately resected and stored at −80°C for use. Rats and mice were maintained in accordance with guidelines of the Institutional Animal Care and Use Committee, and all the animal studies performed were approved by The First Affiliated Hospital of Harbin Medical University Institutional Animal Care and Use Committee.

### Apoptosis Analysis by Annexin V

Apoptosis rate were evaluated at 48 h of culture by Apoptosis kit (Beyotime, China). 1 × 10^6^ cells were washed by PBS and resuspended in 195 ul 1× binding buffer. The cells were treated with 5 μl of fluorochrome conjugated Annexin V for 15 min at room temperature. These cells were washed by PBS and resuspended in 190 ul 1× binding buffer and then 10 μl of propidium iodide solution was added. The fluorescence of stained cells was analyzed by flow cytometry (Becton, Dickinson and Company, Franklin Lakes, NJ, USA) after 1 hour.

### Statistical analysis

All the experiments were repeated three times at least and the statistical results were expressed as mean ± SEM. The significance was evaluated by Student's t-test or one way ANOVA using GraphPad Prism software.

## Author Contributions

S.Z., X.H., Y.L. and H.L. designed research; X.H., H.L., X.C., H.C., F.G., C.W., D.Z., J.W., X.C., C.S., C.L., F.P., Y.B. and Z.Y. performed research; Y.L., G.Y., J.A. and X.G. analyzed data; and X.H., Y.L., H.L. and M.L. wrote the paper. All authors reviewed the manuscript.

## Supplementary Material

Supplementary Information Supplementary information
