# Supplementary material for: PERK silence inhibits glioma cell growth under low glucose stress by blockage of p-AKT and subsequent HK2's mitochondria translocation
Source: Sci Rep. 2015 Mar 12;5:9065. doi: 10.1038/srep09065 (PMC4356960; doi:10.1038/srep09065)
Supplement: Supplementary Information [file srep09065-s1.pdf]

Supplementary information

PERK silence inhibits glioma cell growth under low glucose stress by blockage of p-AKT and subsequent HK2's mitochondria translocation

Xu Hou[1]*, Yaohua Liu[1]*, Huailei Liu[1], Xin Chen[1], Min Liu[2], Hui Che[3], Fei Guo[3], Chunlei Wang[1], Daming Zhang[1], Jianing Wu[1], Xiaofeng Chen[1], Chen Shen[1], Chenguang Li[1], Fei Peng[1], Yunke Bi[1], Zhuowen Yang[4], Guang Yang[1], Jing Ai[3], Xin Gao[5], Shiguang Zhao[1]

*Those authors contributed equally to this work.

[1]Department of Neurosurgery, First Affiliated Hospital of Harbin Medical University, Harbin, Heilongjiang Province, People's Republic of China.

[2]College of Basic Medicine, Beijing University of Chinese Medicine, Beijing, People's Republic of China.

[3]Department of Pharmacology (State-Province Key Laboratories of Biomedicine-Pharmaceutics of China), Harbin Medical University, Harbin, Heilongjiang Province, People's Republic of China.

[4]Department of Endocrinology, First Affiliated Hospital of Harbin Medical University, Harbin, Heilongjiang Province, People's Republic of China.

[5]Computer, Electrical and Mathematical Sciences and Engineering Division, King Abdullah University of Science and Technology (KAUST), Thuwal, Kingdom of Saudi Arabia.

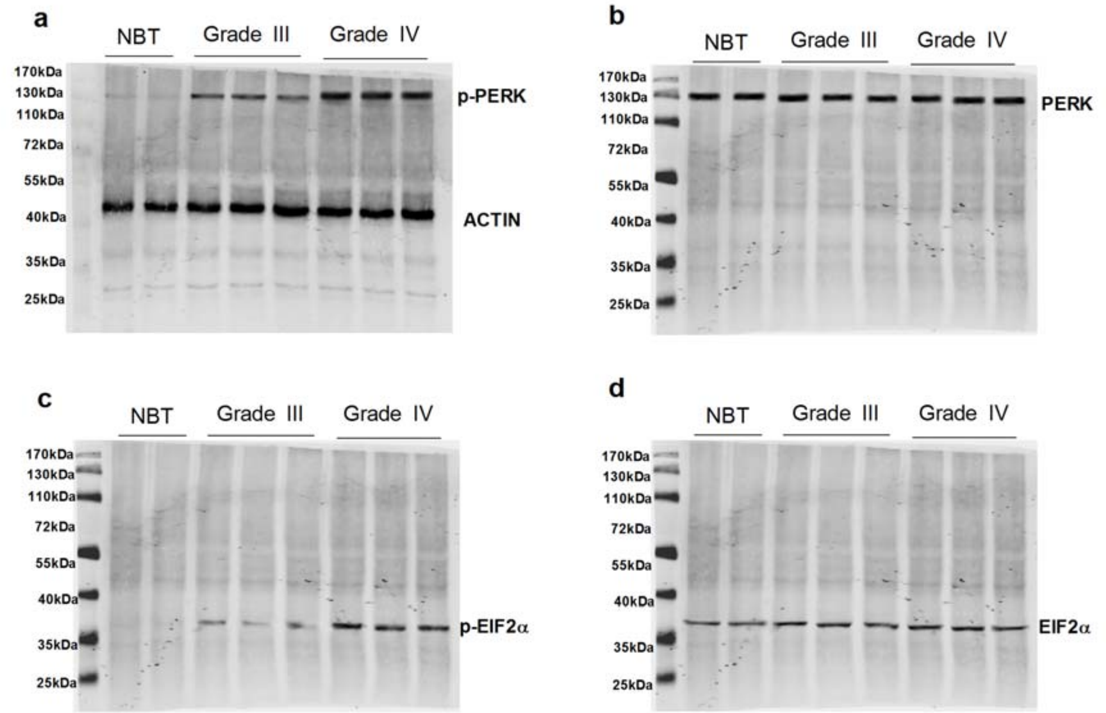

Supplementary figure 1 - PERK is strongly activated in glioma tissues. Westernblot analyses of p-PERK, PERK, p-EIF2α and EIF2α protein expression in different grades of gliomas and nontumor brain tissues (NBT) with the full size panels for immunoblot.

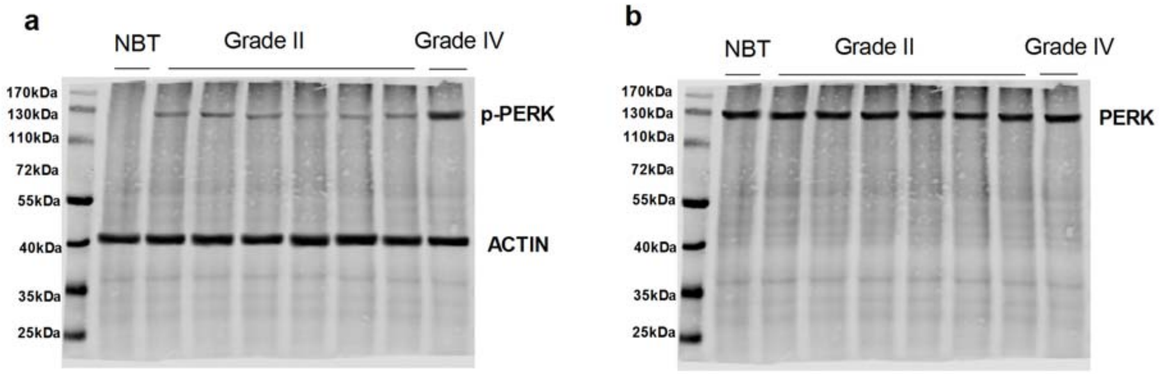

Supplementary figure 2 - PERK is differentially activated in Grade II glioma tissues. Westernblot analyses of p-PERK, PERK, p-EIF2α and EIF2α protein expression in nontumor brain tissue (NBT) sample, 6 Grade II glioma samples and Grade IV grade glioma sample with the full size panels for immunoblot.
